# Problematic Pornography Consumption and Its Associated Factors Among Undergraduate Students of Kathmandu Metropolitan City: A Cross‐Sectional Study

**DOI:** 10.1002/hsr2.71030

**Published:** 2025-07-09

**Authors:** Anisha Chalise, Saloni Pandey, Shishir Paudel, Ram Kumar Chaudhary, Nirmal Raj Marasine

**Affiliations:** ^1^ Center for Research on Environment Health and Population Activities (CREHPA) Lalitpur Nepal; ^2^ Department of Public Health, CiST College Pokhara University Kathmandu Nepal; ^3^ Kathmandu Institute of Child Health Hepali Height Budhanilkantha Nepal; ^4^ Department of Pharmacy, CiST College Pokhara University Kathmandu Nepal

**Keywords:** problematic pornographic consumption, risk pornography consumption, undergraduates, youth

## Abstract

**Background and Aims:**

Problematic pornography consumption has been linked to various physical, mental, and social issues, particularly among young adults. Despite global attention to this issue, limited research exists on its prevalence and contributing factors in Nepal. This cross‐sectional study aimed to assess the factors associated with problematic pornography consumption (PPC) among undergraduate students of Kathmandu Metropolitan City.

**Methods:**

A total of 361 undergraduate students residing in hostels in Kathmandu Metropolitan City were sampled. Data were collected using a self‐administered questionnaire, and problematic pornography consumption was measured using the Problematic Pornography Consumption Scale (PPCS). Bivariate analyses (Chi‐square test and unadjusted odds ratios) and multivariable logistic regression were performed to identify factors associated with PPC, with adjusted odds ratios (aOR) calculated at 95% confidence interval (CI) and 5% level of significance.

**Results:**

Out of 361 undergraduate students, 62 (17.2%; 95% CI: 13.3%–21.3%) students were observed to exhibit PPC. PPC was found to be associated with younger age (< 20 years, aOR: 3.671; 95% CI: 1.546–8.717; 20–22 years, aOR: 2.491; 95% CI: 1.133–5.477), male gender (aOR: 6.178; 95% CI: 2.349–16.244), alcohol consumption (aOR: 2.630; 95% CI: 1.182–5.849), and poor knowledge of risky sexual behavior (aOR: 1.944; 95% CI: 1.042–3.624).

**Conclusion:**

PPC is prevalent among Nepali undergraduate students. The findings highlight the need for gender‐sensitive interventions and educational programs addressing the impacts of pornography on sexual health.

## 1 Background

Pornography is the depiction of sexual content in different media, such as pictures, films, or videos, with the primary intent to bring about sexual excitement or arousal in the consumer [1]. In contemporary society, pornography is disseminated through a range of mediums, including still images, magazines, films, and most commonly, online content [2]. The increasing use of the internet has made pornographic materials more accessible, leading to significant changes in the volume and nature of its consumption over the past decade [3]. This has led to problematic pornography consumption (PPC) habits among consumers, which is often called pornographic addiction, a condition where the person tends to increase the propensity and tendency to consume pornographic content regularly and where its absence leads to psychological distress [4]. It has been suggested that people who consume pornographic content perceive it to be harmless and take it more as educational content to expand their sexual skills [5, 6]. However, many studies have linked pornography consumption to poor sexual health, permissive sexual attitudes, sexual aggression, maladaptive attitudes about relationships, unrealistic attitudes towards sex, objectification of female gender, and stronger gender‐stereotypical sexual beliefs [7, 8, 9].

Problematic pornography use is characterized by the persistent inability to control the urge to consume pornographic content, despite its detrimental impact on physical, mental, and social well‐being [10]. It has been considered a form of Compulsive Sexual Behavior Disorder in the 11th edition of the International Statistical Classification of Diseases and Related Health Problems (ICD‐11) [11]. Studies have shown that adolescent pornography use has often been associated with stronger gender‐stereotypical sexual beliefs and stronger permissive sexual attitudes with the probability of the occurrence of risky sexual behaviors [9]. Pornography consumption has been observed to be common among males, especially in the young age group, and decreases with the increase in age [12]. In countries such as Australia, North America, and Europe, between 70% and 94% of adults report lifetime pornography use, with rates of problematic consumption ranging from 3% to 38% [13, 14, 15, 16, 17]. Similarly, in the context of Asia, the prevalence of PPC among undergraduate medical students in India was found to be between 12.5% and 14.6% [18, 19]. Pornographic use has increased across cultures from conservative countries to modern ones, consequently increasing the problematic pornographic use, highlighting the need for further studies on it [20].

In the context of Nepal, at present, watching pornographic content in private is not a criminal offense; however, there has been a ban on sharing, distribution, and broadcasting of pornographic content through any medium since 2018, making its distribution a criminal offense [21]. The government took this decision in response to the increasing cases of rape and sexual harassment in the country, but there have been conflicting arguments that the ban could result in more sexual violence [21, 22]. Previous studies have estimated that between 52.4% and 62.0% of Nepalese youth and adolescents have viewed pornographic content [23, 24, 25]. Despite the ban on distribution of pornographic content, over 1011 cases of revenge pornography have been reported to Nepal's cyber bureau in 2022/23, revealing gaps in the legal framework to combat this cybercrime [26]. To our knowledge, no studies have specifically explored the prevalence and factors contributing to PPC among this vulnerable adolescent and youth population of Nepal. In 2023, a study among hostel students in Kathmandu Metropolitan City sought to assess risky sexual behaviors, including PPC as an independent factor [27]. This study uses secondary data derived from the same data set collected by Chaudhary et al. (2024) and aimed to assess the factors contributing to PPC among Nepali youth using secondary data.

### 1.1 Research Question

What are the factors associated with PPC among undergraduate students residing in hostels in Kathmandu Metropolitan City?

## 2 Methods

### 2.1 Study Design and Participants

This study is based on a secondary analysis of a cross‐sectional survey conducted among undergraduate students residing in hostels of Kathmandu Metropolitan, between July and October 2023, to investigate their risky sexual behavior [27]. The primary study by Chaudhary et al. (2024) focused broadly on factors associated with risky sexual behaviors. In contrast, the present analysis addresses a distinct research question, aiming to identify the factors specifically associated with PPC. For this purpose, only relevant variables from the original data set were extracted and reanalyzed. The undergraduate students residing in the sampled hostels for at least 6 months before data collection were eligible participants for the study.

### 2.2 Sample Size

The sample size was calculated using the formula, *n* = (*NZ* ^2^ *pq*)/(*d* ^2^(*N *− 1) + *Z* ^2^ *pq*) where, *z* = standard normal variate, with a value of 1.96 at 95% Confidence Interval (CI), *p* = prevalence of risky sexual behavior at 73% among school‐going students of rural Nepal aged 15–24 years [28], q = 1 − *p* and *d* = allowable error (5%), *N* = 6060 (total number of undergraduates residing in the hostels of Kathmandu Metropolitan). The sample size was then adjusted for a 30% nonresponse rate, considering the sensitivity of the research problem. This yielded a sample size of 373. A two‐stage random sampling strategy was employed for the sampling of 373 participants. In the first stage, from a total of 196 hostels in Kathmandu Metropolitan City that agreed to participate in the study, 20 hostels were randomly selected using STATS V.2.0 software. In the second stage, 18–19 undergraduate students residing in each of these selected hostels were chosen randomly. Proportional stratification based on the academic discipline or socioeconomic status of the participants was not possible due to the lack of background information on hostel residents during the development of the sampling frame.

### 2.3 Data Collection

Data were collected using a self‐administered questionnaire, distributed, and collected in a sealed envelope to protect participants' anonymity. All the students were oriented about the questions presented in the questionnaire for clarification, and written informed consent was acquired from them before handing them the enclosed questionnaire.

### 2.4 Outcome Variable

The primary outcome variable for this secondary analysis was PPC assessed through PPC Scale (PPCS‐18) [29]. The original PPCS‐18 provided a cutoff of > 75 for pornography consumption to be considered problematic consumption [29]. However, based on the characteristics of the sample distribution, an additional intermediate “at‐risk” category was introduced to reflect the PPCS scores ranging from 50 to 75 [27]. This categorization aimed to capture individuals exhibiting elevated but sub‐threshold consumption patterns. The individuals with PPCS scores < 50 were categorized as exhibiting low pornography consumption (non‐PPC), scores ranging from 50 to 75 were categorized as being at risk of problematic consumption (elevated consumption), and scores > 75 were categorized as exhibiting high pornography consumption. For analysis, the individuals with PPCS scores ≥ 50 were grouped as exhibiting PPC [27].

The knowledge of risky sexual behavior was assessed using a self‐developed questionnaire, designed after an extensive literature review and consultation with six experts in the field of public health and reproductive health to ensure face and content validity. The questionnaire included items on modes of STI transmission, STI symptoms, condom effectiveness, consequences of unprotected sex, and preventive measures of STI. Responses were scored as correct (1) or incorrect (0), and a total knowledge score was computed for each participant. A median split was applied to categorize the scores into “poor” and “good” knowledge levels. The internal consistency of the knowledge scale was acceptable, with a Cronbach's alpha of 0.78. Variables such as peer pressure for sexual relationships and involvement with commercial sex workers were assessed using single‐item, binary (yes/no) self‐reported questions.

### 2.5 Data Processing, Management, and Analysis

Data were entered in EpiData 3.1 and exported to Statistical Package for the Social Sciences V.20 for statistical analysis. The data were summarized in terms of frequency, percentage, mean, and standard deviation. The Chi‐square test, and unadjusted odds ratio (uOR) with 95% CI were calculated. A two‐sided test with a significance level of 5% was applied to identify the factors associated with PPC. The factors found to have statistical significance (*p *< 0.05) in the Chi‐square test were then subjected to the final model of multivariable logistic regressions for adjusted odds ratio (aOR). Before performing multivariable analysis, the multi‐collinearity among selected independent variables was tested using the variance inflation factor (VIF), where a VIF greater than five was taken as an indication of multi‐collinearity between the independent variables.

## 3 Results

Out of the total of 373 undergraduates approached for the study, 361 provided complete responses. Of the 361 participants, slightly more than half were male (57%), and most came from nuclear families (59%). Participants' ages ranged from 18 to 32 years, with a mean age of 21.7 ± 2.72 years. Nearly a third (30%) reported being in a relationship at the time of the study. The largest academic group was management students (30%), followed closely by health science students (29%). Regarding reproductive health, just over half (54%) of the participants demonstrated good knowledge of risky sexual behavior. Peer pressure to engage in sexual relationships was reported by nearly a third of the participants (30%). More than a fourth (29%) of the participants were current consumers of alcohol (within the past 6 months), and 17% were current smokers (within the past 6 months). About two‐fifths of the participants (38%) were sexually active, and of those, 28% reported having multiple sexual partners. Additionally, out of 361 participants, 14% of them reported engaging with commercial sex workers within the past year (Table 1).

**Table 1:** General characteristics of the participants.

Characteristics	*n* (%)
**Age**	
< 20 years	133 (36.8)
20–25 years	178 (49.3)
≥ 25 years	50 (13.9)
**Gender**	
Male	206 (57.1)
Female	155 (42.9)
**Family type**	
Nuclear	213 (59.0)
Joint/extended	148 (41.0)
**Permanent residency**	
Urban	98 (27.1)
Semi‐urban	193 (53.5)
Rural	70 (19.4)
**Relationship status**	
Never been in a relationship	160 (44.3)
Currently in a relationship	108 (29.9)
Been in a relationship in the past	93 (25.8)
**Academic discipline**	
Health Science	104 (28.8)
BSc Science	31 (8.6)
Management	108 (29.9)
Engineering	42 (11.6)
Education/Humanities	76 (21.1)
**Peer pressure for a sexual relationship**	
Absence	254 (70.4)
Presence	107 (29.6)
**Smoking status**	
Nonsmoker	299 (82.8)
Smoker	62 (17.2)
**Alcohol consumption**	
Nonconsumer	256 (70.9)
Consumer	105 (29.1)
**Knowledge of risky sexual behavior**	
Poor knowledge	166 (46.0)
Good knowledge	195 (54.0)
**Involvement in a sexual relationship**	
Yes	136 (37.7)
No	225 (62.3)
**Number of current sexual partners (*n* = 136)**	
Single sexual partner	98 (72.1)
Multiple sexual partners	38 (27.9)
**Involved in sex with a commercial sex worker**	
Yes	49 (13.6)
No	312 (86.4)

**Figure 1:**
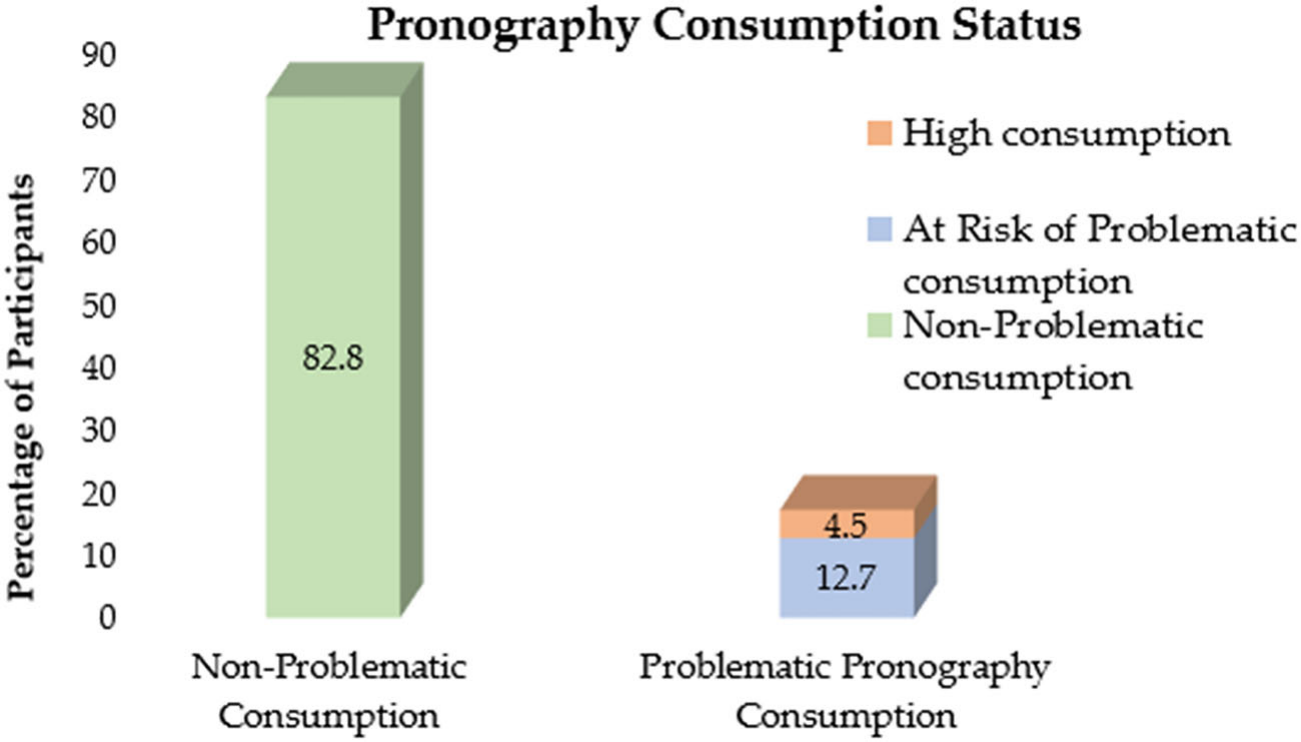
Status of pornography consumption among participants.

The total PPCS‐18 score ranged from 18 to 114, with a mean score of 31.38 ± 19.13. The median score was 21, and the interquartile range was 22. The distribution of scores was positively skewed (Skewness = 1.712; Kurtosis = 2.700), indicating that most individuals reported lower levels of pornography consumption. The detailed responses of the participants on the PPCS‐18 tool are provided in Supporting Information S1: Table 1. A total of 299 participants (82.8%, 95% CI: 77.9%–86.9%) had non‐PPC, defined by a PPCS score below 50. Forty‐six participants (12.7%, 95% CI: 9.4%–16.6%) were categorized as being at risk of PPC, with PPCS scores ranging from 50 to 75. Sixteen participants (4.5%, 95% CI: 2.6%–7.3%) had high pornography consumption, defined by a score >75. The scores ranging between 50 and 75 represent those individuals who are on a trajectory toward problematic consumption. A total of 62 participants (17.2%, 95% CI: 13.5%–20.9%) were categorized as having PPC, combining the individuals at risk (score 50–75) and those with high pornography consumption (score > 75) (Figure 1).

In bivariate analysis, participants' age and gender were the socio‐demographic factors found to be associated with PPC. Similarly, lifestyle and psychological factors like peer pressure for a sexual relationship, current smoking status, current alcohol consumption, knowledge of risky behavior, involvement in sexual relationships, number of current sexual partners, and involvement in sex with a commercial sex worker were found to have a statistically significant relationship with PPC (Table 2).

**Table 2:** Association of participants' characteristics with PPC.

	PPC		*χ* ^2^	*p* value
Characteristics	Presence *n* (%)	Absence *n* (%)		
**Age**				
< 20 years	32 (24.1)	101 (75.9)	6.852	0.033*
20–25 years	23 (12.9)	155 (87.1)		
≥ 25 years	7 (14.0)	43 (86.0)		
**Gender**				
Male	56 (27.2)	150 (72.8)	33.795	< 0.001*
Female	6 (3.9)	149 (96.1)		
**Family type**				
Nuclear	31 (14.6)	182 (85.4)	2.508	0.113
Joint/extended	31 (20.9)	117 (79.1)		
**Permanent residency**				
Urban	14 (14.3)	84 (85.7)	1.221	0.543
Semi‐urban	37 (19.2)	156 (80.8)		
Rural	11 (15.7)	59 (84.3)		
**Relationship status**				
Never been in a relationship	26 (16.2)	134 (83.8)	0.945	0.623
Currently in relationship	17 (15.7)	91 (84.3)		
Been in a relationship in the past	19 (20.4)	74 (79.6)		
**Academic discipline**				
Health Science	13 (12.5)	91 (87.5)	2.720	0.606
BSc Science	5 (16.1)	26 (83.9)		
Management	21 (19.4)	87 (80.6)		
Engineering	9 (21.4)	33 (78.6)		
Education/Humanity	14 (18.4)	62 (81.6)		
**Peer pressure for a sexual relationship**				
Absence	28 (11.0)	226 (89.0)	22.792	<0.001*
Presence	34 (31.8)	73 (68.2)		
**Smoking status**				
Nonsmoker	40 (13.4)	259 (86.6)	17.641	<0.001*
Smoker	22 (35.5)	40 (64.5)		
**Alcohol consumption**				
Nonconsumer	30 (11.7)	226 (88.3)	18.417	<0.001*
Consumer	32 (30.5)	73 (69.5)		
**Knowledge of risky sexual behavior**				
Poor Knowledge	36 (21.7)	130 (78.3)	4.399	0.036*
Good Knowledge	26 (13.3)	169 (86.7)		
**Involvement in a sexual relationship**				
Yes	32 (23.5)	104 (76.5)	6.195	0.013*
No	30 (13.3)	195 (86.7)		
**Number of current sexual partners (*n* = 136)**				
Single sexual partner	17 (17.3)	81 (82.7)	7.451	0.006*
Multiple sexual partners	15 (39.5)	23 (60.5)		
**Involved in sex with a commercial sex worker**				
Yes	17 (34.7)	32 (65.3)	12.233	<0.001*
No	45 (14.4)	267 (85.6)		

* Significance at *p *< 0.05

For multivariate analysis, the VIF test was performed among the independent variables, which were found to have a statistically significant relationship with PPC in bivariate analysis. The highest reported VIF was 1.749, confirming no issue of multi‐collinearity. It was observed that, compared to the undergraduate students aged above 22 years, those aged less than 20 years were three times more at odds (aOR: 3.671; 95% CI: 1.546–8.717) and those aged between 20 and 22 years were twice more at odds (aOR: 2.491; 95% CI: 1.133–5.477) of exhibiting PPC. Similarly, males were observed to have six times higher odds (aOR: 6.178; 95% CI: 2.349–16.244) of exhibiting PPC than females. Current alcohol consumers had a two‐fold increased odds (aOR: 2.630; 95% CI: 1.182–5.849) of exhibiting PPC as compared to non‐consumers. In comparison to the participants with good knowledge of risky sexual behavior, those with poor knowledge were almost twice more at odds (aOR: 1.944; 95% CI: 1.042–3.624) of exhibiting PPC. The participants who had sex with a commercial sex worker had higher odds of exhibiting PPC (aOR: 1.541; 95% CI: 1.270–3.695). Notably, bivariate analysis illustrated that participants with peer pressure for sexual relationships were three times more at odds (uOR: 3.759; 95% CI: 2.136–6.618) of exhibiting PPC. However, this association weakened and became nonsignificant after accounting for other factors in the adjusted model (aOR: 1.859; 95% CI: 0.934–3.701) (Table 3).

**Table 3:** Factors associated with PPC.

Variables	PPC			
	uOR (95% CI)	*p* value	aOR (95% CI)	*p* value
**Age**				
< 20 years	2.949 (1.383–6.285)	0.005*	3.671 (1.546–8.717)	0.003*
20–22 years	2.047 (1.011–4.145)		2.491 (1.133–5.477)	0.023*
> 22 years	Ref		Ref	
**Gender**				
Male	9.271 (3.877–22.172)	< 0.001*	6.178 (2.349–16.244)	<0.001*
Female	Ref		Ref	
**Peer pressure for a sexual relationship**				
Absence	Ref		Ref	
Presence	3.759 (2.136–6.618)	< 0.001*	1.859 (0.934–3.701)	0.870
**Smoking status**				
Nonsmoker	Ref		Ref	
Smoker	3.561 (1.920–6.605)	< 0.001*	1.070 (0.870–2.071)	0.694
**Alcohol consumption**				
Nonconsumer	Ref		Ref	
Consumer	3.302 (1.879–5.802)	< 0.001*	2.630 (1.182–5.849)	0.018*
**Knowledge of risky sexual behavior**				
Poor Knowledge	1.800 (1.035–3.132)	0.038*	1.944 (1.042–3.624)	0.037*
Good Knowledge	Ref		Ref	
**Involvement in a sexual relationship**				
Yes	2.001 (1.152–3.474)	0.014*	1.230 (0.425–3.561)	0.703
No	Ref		Ref	
**Number of current sexual partners (*n* = 136)**				
Single sexual partner	Ref		Ref	
Multiple sexual partners	3.170 (1.349–7.159)	0.008*	1.956 (0.711–5.378)	0.194
**Had sex with a commercial sex worker**				
Yes	3.152 (1.617–6.146)	< 0.001*	1.541 (1.270–3.695)	0.043*
No	Ref			

Abbreviations: aOR, adjusted odds ratio; uOR, unadjusted odds ratio.

* Significance at *p*< 0.05

## 4 Discussion

This study assessed the prevalence of PPC and its associated factors among Nepali youth. It was observed that nearly one in five undergraduate students exhibited PPC. In the context of Nepal, few studies have assessed the rate of pornography consumption among adolescents and youth, but there are no prior studies assessing problematic consumption among Nepali youth. Past studies suggested that the prevalence of pornography consumption among Nepali youth lies between 52.4% and 62.0% [23, 24, 25]. Similarly, the prevalence of pornography exposure among Nepalese adolescents lies between 55.4% and 62.0% [23, 24]. A higher rate of PPC has been observed among students throughout the world, marking it as one of the serious problems. In the neighboring country India, a study conducted in 2020 among 1926 undergraduate medical students reported that 14.6% (95% CI: 12.4%–16.1%) of the undergraduates exhibit PPC [19]. Similarly, a study from Poland suggested that 80% of students have pornographic exposure, with 15.5% stating a self‐perceived pornographic addiction [30]. Globally, pornography is becoming common in the adolescent and young population and is currently a part of the sexuality of pre‐adolescents, adolescents, and adults [3]. Young people often use sexually explicit content to stimulate themselves and satiate their curiosity about nudity and sexual activity [31]. Internet technology has become more accessible, affordable, and anonymous, facilitating individuals to access online materials of a sexual nature, including online pornography materials [32, 33]. These can be the major reasons for increasing pornography consumption and addiction among youth worldwide.

This study showed that students below the age of 22 years are more likely to exhibit PPC. This is consistent with the past study from India, where it was observed that participants having an early age of exposure to pornography tend to have a higher score on the PPCS [18]. Similarly, the studies based in Kenya and Poland stated the median age of first exposure to pornography lies between the ages of 13–18 years, and this age was associated with an increased likelihood of negative effects from pornography in young adults [34, 35]. It is important to note that exposure to pornography from earlier ages may affect the psychosocial development in childhood and adolescence, which could further lead to an adverse outcome on social relationships, mental health, and sexual performance [30].

It was observed that males were six times more likely to exhibit PPC than females. This is consistent with findings shared from India, where males demonstrated a higher likelihood of problematic pornography use as compared to female counterparts [18, 19]. A higher rate of consumption of pornographic content among males was also reported by another study in the neighboring country, China [36]. Similar observations were shared by studies throughout the world, such as a study based on Italian students reported that 89% of males and 39% of females were exposed to pornographic materials [37]. Similarly, in the United States, 91.5% of males and 60.2% of females were involved in pornography consumption [38]. These findings are in line with different studies throughout the world illustrating the higher engagement of males in pornographic content [39, 40, 41, 42, 43]. Furthermore, an international sex survey conducted in 42 countries noted that men had the highest problematic pornographic use scores than any other gender [44]. These findings highlight the need for gender‐sensitive intervention to limit males' exposure to pornographic content.

It was observed that a statistically significant relationship exists between alcohol use and PPC, as those who currently consumed alcohol were twice more likely to exhibit PPC. This is consistent with a past study from Nepal, where it was observed that students who consume alcohol were more exposed to pornographic content regularly and were more prone to risky sexual behavior [24]. Similarly, a study among Italian students noted increased exposure to violent or degrading pornography during alcohol consumption [37]. Alcohol consumption and pornographic exposure are not only correlated, but they have also been linked to risky sexual activities, including engagement in sexual relationships with multiple partners [45].

Students who were observed to have a poor level of knowledge regarding risky sexual behavior were found to be more engaged in pornography consumption. This is consistent with a past study from Nepal where students with reproductive health education were found to have lower odds of being engaged in risky sexual behavior including pornography consumption [24]. It has been suggested that exposure to explicit sexual media can develop promiscuous sexual attitudes that are strongly linked with dangerous sexual actions [39]. However, many consumers first seek information about sex and sexuality from pornography due to their curiosity, as well as limited access to proper sex education materials and no prior sexual exposure, and use it as a means to teach themselves, considering it to be a safer approach [46, 47]. But in fact, higher exposure to pornographic content could lead to risky sexual behavior.

In the unadjusted analysis, it was found that those who were involved in sexual relationships, and engaged with multiple sexual partners had twice or three times the odds of exhibiting PPC, but this relation weakened and became insignificant in the adjusted model while considering other factors. Likewise, it was also observed that PPC was higher among those who reported involving in sexual activities with commercial sex workers. These findings are somehow supported by past studies where exposure to pornography was linked with a higher likelihood of multiple lifetime sexual partners and higher involvement in sexual activities under substance use [48]. These observations suggested that a complex interaction exists between pornography consumption and different risky sexual behaviors and multiple other factors should be considered while making any assumptions. All of these findings suggest a need for tailored pornography education programs to educate students on the adverse effects of pornography, with targeted treatment programs for sexual addiction, sexual abuse, and pornography abuse [49].

This is the first study to report the prevalence of PPC among Nepalese undergraduate students. With a combined 17.2% of students exhibiting at‐risk or high pornography consumption, our findings provide important baseline data for the South Asian region. The findings confirm well‐established correlates of PPC with male gender, younger age, and alcohol use, reinforcing their cross‐cultural consistency while also highlighting culturally specific risk factors within the Nepalese context. These insights carry important policy implications, emphasizing the need for gender‐sensitive mental health interventions targeted toward male students, as well as the integration of comprehensive sexuality education into university curricula to bridge knowledge‐behavior gaps and mitigate the risks associated with PPC.

Despite these contributions of this study, several limitations must be acknowledged. First, the sensitive nature of the topic and reliance on self‐reported data may have led to social desirability bias, with participants possibly underreporting certain behaviors. To minimize this risk, we ensured confidentiality by using sealed, self‐administered questionnaires and creating a private environment during data collection to encourage more honest responses. While these measures were designed to reduce bias, the possibility of some underreporting cannot be completely ruled out. Second, although random sampling was employed at both the hostel and participant levels, proportional stratification by academic discipline or socioeconomic status was not feasible due to the unavailability of relevant background information in the initial planning of the sampling procedure. Therefore, while students from various academic disciplines were included, the representativeness of the sample may be limited. Third, the study was confined to students residing in hostels within Kathmandu Metropolitan City, which may not fully capture the broader cultural, ethnic, and regional diversity of Nepal. Fourth, while subgroup analyses provided additional insights, the relatively small sample sizes within certain subgroups may have limited statistical power. As such, findings from these analyses should be interpreted with caution and warrant validation in studies with larger and more diverse samples. Finally, we acknowledge that the use of an intermediate “at‐risk” category (PPCS score 50–74) is exploratory. This decision was made to capture elevated but sub‐threshold patterns of pornography consumption that may warrant attention. However, this categorization has not yet been validated, and future research should assess its psychometric robustness and clinical relevance in similar populations. Further research with a larger and more geographically representative sample is recommended across different regions and cultures.

## 5 Conclusion

This study highlights the significant prevalence of PPC among undergraduate students in Nepal and identifies key factors such as age, gender, alcohol consumption, and knowledge of risky sexual behaviors, which are associated with it. The findings emphasize the importance of addressing these factors through targeted interventions and educational programs. The findings highlight the need for gender‐sensitive interventions and educational programs addressing the impacts of pornography on sexual health.

## Author Contributions

**Anisha Chalise:** conceptualization, methodology, validation, supervision, visualization, writing – original draft, writing – review and editing, formal analysis, project administration, data curation, software. **Saloni Pandey:** data curation, investigation, formal analysis, writing – review and editing. **Shishir Paudel:** conceptualization, methodology, data curation, investigation, validation, formal analysis, supervision, visualization, writing – original draft, writing – review and editing. **Ram Kumar Chaudhary:** resources, investigation, data curation, funding acquisition, project administration. **Nirmal Raj Marasine:** writing – review and editing, formal analysis, visualization.

## Ethics Statement

This study involves human participants and adheres to the ethical principles outlined in the Declaration of Helsinki regarding the use of human subjects in research. The ethical approval for this study was obtained from the institutional review committee of CiST College (Ref no. 47/080/081). Written informed consent was obtained from each participant before data collection, where they were informed about the possibility of future anonymized analyses of the collected data. The questionnaire was distributed to and collected back from participants in a closed envelope, to maintain the confidentiality of participants' information. To ensure privacy, the participants were requested to fill out the questionnaire in an empty room of their hostel.

## Conflicts of Interest

The authors declare no conflicts of interest.

## Transparency Statement

The corresponding author, Shishir Paudel, affirms that this manuscript is an honest, accurate, and transparent account of the study being reported; that no important aspects of the study have been omitted; and that any discrepancies from the study as planned (and, if relevant, registered) have been explained.

## Supporting information

Supplementary Table.

## Data Availability

The data that support the findings of this study are available from the corresponding author upon reasonable request.
