# Supplementary material for: Problematic Pornography Consumption and Its Associated Factors Among Undergraduate Students of Kathmandu Metropolitan City: A Cross‐Sectional Study
Source: Health Sci Rep. 2025 Jul 9;8(7):e71030. doi: 10.1002/hsr2.71030 (PMC12241428; doi:10.1002/hsr2.71030)
Supplement: Supplementary file 1 — Supplementary Table. [file HSR2-8-e71030-s001.docx]

**Pornography Consumption Status**

The total PPCS-18 scores ranged from 18 to 114, with a mean score of 31.38 ± 19.13. The median score was 21, and the interquartile range (IQR) was 22. It was observed that 83% of participants fell under the non-problematic consumption category, while 13% were at risk of developing problematic consumption, and 4% exhibited problematic consumption. Among the 361 undergraduates, those at risk of developing problematic consumption and those with high consumption were collectively categorized as having PPC. This category was found to encompass 17% of participants (95% CI: 13.3%-21.3%), equating to 62 individuals identified as experiencing problematic pornography consumption (PPC). The detailed responses of the participants on the PPCS-18 tool are provided in Supp-Table 1.

Supp Table 1: Response of the participant in PPCS-18

| **Characteristics** | **Never** | **Rarely** | **Occasionally** | **Sometimes** | **Often** | **Very Often** | **All the Time** |
| --- | --- | --- | --- | --- | --- | --- | --- |
| Felt porn is an important part of life | 206 (57.1) | 56 (15.5) | 21 (5.8) | 53 (14.7) | 10 (2.8) | 6 (1.7) | 9 (2.5) |
| Use porn to restore tranquillity of feelings | 225 (62.3) | 61 (16.9) | 17 (4.7) | 34 (9.4) | 20 (5.5) | 2 (0.6) | 2 (0.6) |
| Felt porn cause problems in sexual life | 258 (71.5) | 32 (8.9) | 11 (3.0) | 31 (8.6) | 14 (3.9) | 7 (1.9) | 8 (2.2) |
| Felt watching more porn resulted in satisfaction | 259 (71.7) | 40 (11.1) | 17 (4.7) | 19 (5.3) | 15 (4.2) | 3 (0.8) | 8 (2.2) |
| Unsuccessful attempt at reducing the frequency of watching porn | 262 (72.6) | 32 (8.9) | 15 (4.2) | 23 (6.4) | 11 (3.0) | 4 (1.1) | 14 (3.9) |
| Became stressed when being prevented from watching porn | 293 (81.2) | 23 (6.4) | 14 (3.9) | 16 (4.4) | 5 (1.4) | 3 (0.8) | 7 (1.9) |
| Thought about how good watching porn would be | 221 (61.2) | 54 (15.0) | 24 (6.6) | 32 (8.9) | 15 (4.2) | 5 (1.4) | 10 (2.8) |
| Felt watching porn help rid negative feelings | 244 (67.6) | 39 (10.8) | 11 (3.0) | 37 (10.2) | 13 (3.6) | 9 (2.5) | 8 (2.2) |
| Felt watching porn prevented them from bringing out their best | 279 (77.3) | 22 (6.1) | 11 (3.0) | 18 (5.0) | 10 (2.8) | 9 (2.5) | 12 (3.3) |
| Felt they needed more porn to satisfy their needs | 285 (78.9) | 34 (9.4) | 12 (3.3) | 13 (3.6) | 7 (1.9) | 5 (1.4) | 5 (1.4) |
| Kept vow of not watching porn only for a short period | 260 (72.0) | 25 (6.9) | 21 (5.8) | 19 (5.3) | 17 (4.7) | 7 (1.9) | 12 (3.3) |
| Became agitated when unable to watch porn | 291 (80.6) | 23 (6.4) | 15 (4.2) | 12 (3.3) | 5 (1.4) | 11 (3.0) | 4 (1.1) |
| Planned continually on when to watch porn | 281 (77.8) | 29 (8.0) | 20 (5.5) | 13 (3.6) | 8 (2.2) | 2 (0.6) | 8 (2.2) |
| Released tension by watching porn | 253 (70.1) | 33 (9.1) | 25 (6.9) | 23 (6.4) | 7 (1.9) | 8 (2.2) | 12 (3.3) |
| Neglected other leisure activities as a result of watching porn | 270 (74.8) | 25 (6.9) | 21 (5.8) | 21 (5.8) | 15 (4.2) | 5 (1.4) | 4 (1.1) |
| Gradually watched more “extreme” pornographic content after feeling that previous content was less satisfactory | 267 (74.0) | 25 (6.9) | 19 (5.3) | 21 (5.8) | 8 (2.2) | 6 (1.7) | 15 (4.2) |
| Resisted watching porn only for a little while before relapsing | 244 (67.6) | 33 (9.1) | 13 (3.6) | 25 (6.9) | 22 (6.1) | 11 (3.0) | 13 (3.6) |
| Missed porn greatly if not watched for a while | 280 (77.6) | 35 (9.7) | 9 (2.5) | 15 (4.2) | 6 (1.7) | 6 (1.7) | 10 (2.8) |
